# A rare case of meningeal carcinomatosis and internal auditory canal metastasis presenting with the deafness for gastric cancer

**DOI:** 10.1186/s40792-020-01018-1

**Published:** 2020-11-23

**Authors:** Takayuki Tanaka, Kengo Kanetaka, Takahiro Ikeda, Shun Yamaguchi, Syunsuke Kawakami, Tomoo Kitajima, Toru Iwata, Susumu Eguchi

**Affiliations:** 1 Department of Surgery, Nagasaki Rosai Hospital, 2-12-5 Setogoshi, Zip code 857-0134, Sasebo, Nagasaki Japan; 2 Department of Surgery, Nagasaki University Graduate School of Biomedical Sciences, 1-7-1 Sakamoto, Zip code, 852-8501, Nagasaki, Nagasaki Japan

**Keywords:** Meningeal carcinomatosis, Gastric cancer, Bilateral deafness, Internal auditory canal metastasis

## Abstract

**Background:**

Meningeal carcinomatosis is a very rare metastatic site of gastric cancer and meningeal carcinomatosis without other metastatic sites is much extremely rare. Herein, we report our experience with a very rare case of meningeal carcinomatosis which was difficult to diagnose the recurrence by general systemic examination and was found due to the deafness despite the sustained high tumor markers.

**Case presentation:**

A 68-year-old man consulted a hospital with vomiting and hematemesis. Laboratory tests revealed severe anemia. He was referred to our hospital and underwent an emergency gastroscopy, which revealed Borrman type 3 tumor and oozing of blood. Biopsy specimen showed gastric cancer. After several examinations, total gastrectomy was performed and tegafur-gimeracil-oteracil potassium (S-1) was initiated as adjuvant chemotherapy one month after surgery. Tumor marker levels (CEA and CA19-9) remained high for three months after surgery. S-1 was continued while shortening the imaging study follow-up period. Nine months after surgery, he noticed difficulty in hearing with facial paralysis, dizziness, tinnitus, and appetite loss. He was diagnosed with meningeal carcinomatosis and bilateral internal auditory canal metastasis. He died approximately two months later.

**Conclusion:**

Meningeal carcinomatosis should be considered if bilateral deafness and vestibulopathy develop after gastrectomy, even if no recurrence is apparent in the abdominal cavity.

## Background

Gastric cancer is the fourth malignancy and the second cause of the cancer-related death globally [1, 2]. Although, patients with early gastric cancer can be cured by surgical resection, patients with advanced gastric cancer sometimes relapse after curative resection or are initially diagnosed with metastatic diseases. The most common metastatic sites of gastric cancer are the liver, peritoneum, lymph nodes, lung, and bone [3]. Meningeal carcinomatosis is an extremely rare metastatic site of gastric cancer, which occur in 0.1–0.69% of patients [4–7]. Meningeal carcinomatosis is often accompanied by brain parenchymal metastasis, leptomeningeal carcinomatosis, and distant metastasis in other sites [8]. Therefore, meningeal carcinomatosis without other metastases is very rare. Furthermore, bilateral deafness and vestibulopathy due to bilateral internal auditory canal (IAC) metastasis from gastric cancer is extremely rare [8]. Primary and metastatic brain tumor, neurofibromatosisII, Guillain-Barré syndrome, Fisher syndrome among others, may cause gradual or sudden bilateral loss of hearing and vestibular function [9]. We report a rare and suggestive clinical case presenting sudden deafness and facial paralysis with a rapid progress due to metastatic recurrences from gastric cancer.

## Case presentation

**Fig. 1:**
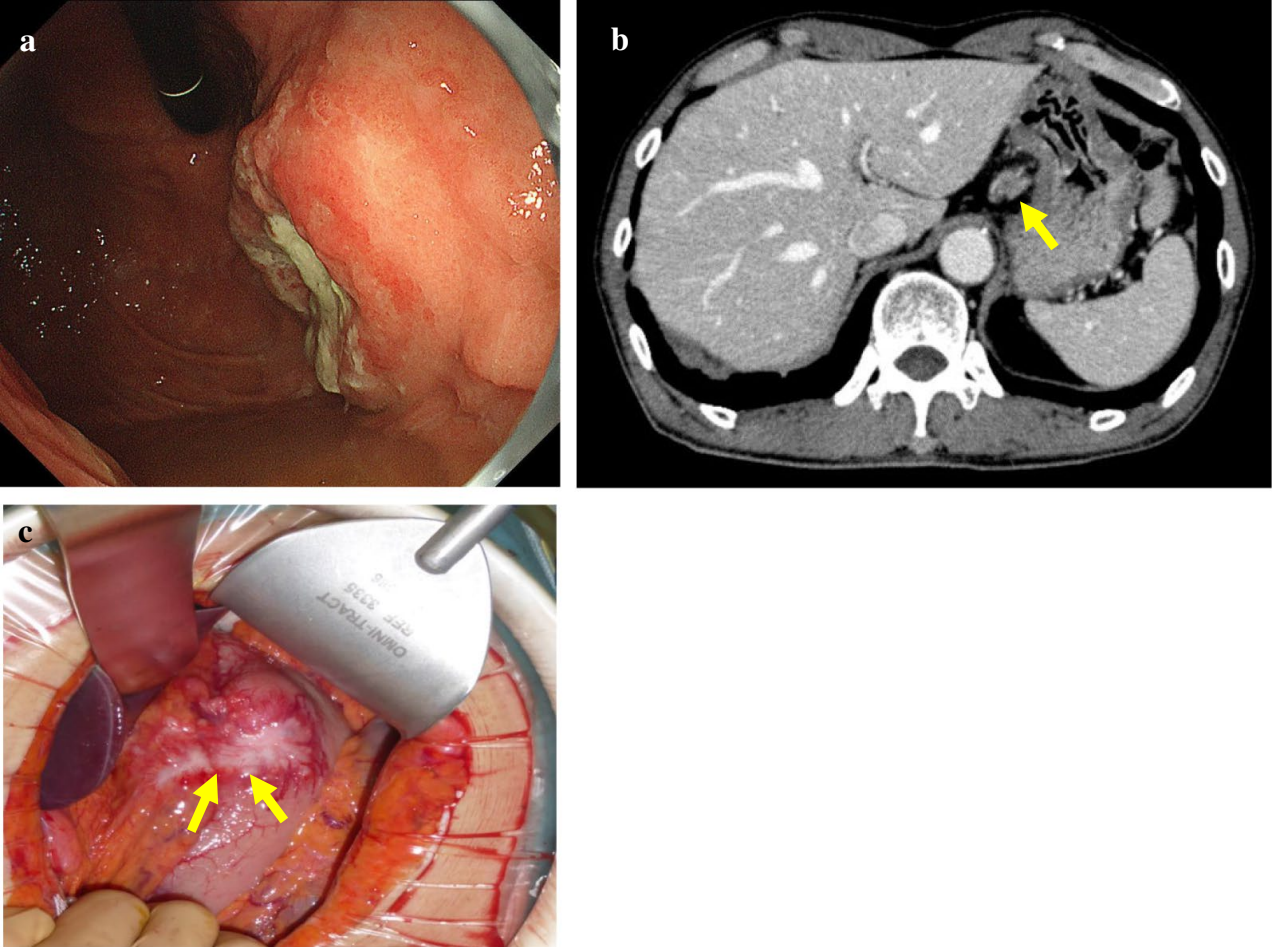
Preoperative and intraoperative findings and the specimen. **a** GS revealed a 3-cm sized mass on the anterior wall of the lesser curvature of the gastric body. **b** CT scan revealing a thick wall of the stomach and the larger lymph nodes in the perigastric area (yellow arrow). **c** The tumor was exposed outside the serosa and was observed to have progressed to the lesser omentum

**Fig. 2:**
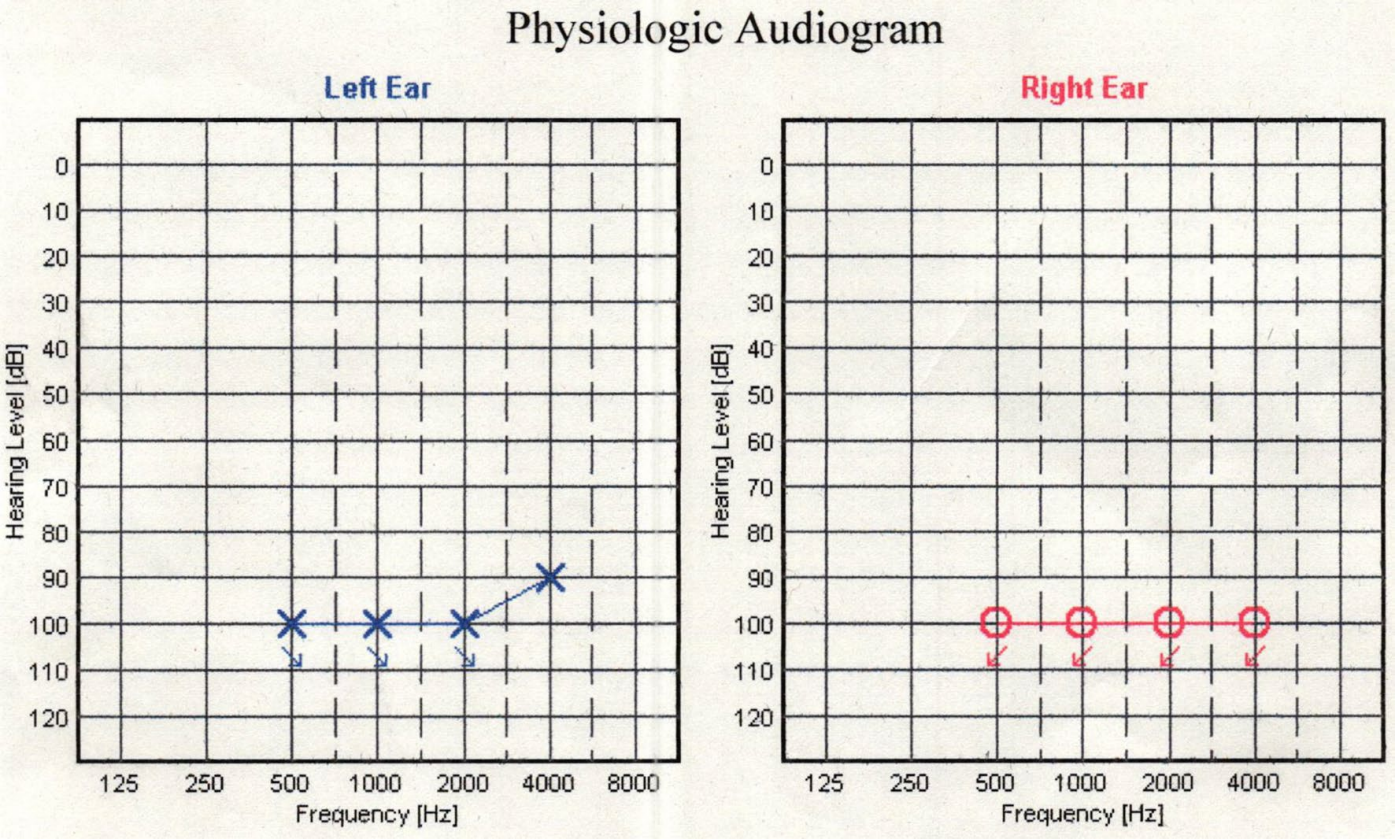
Physiological audiogram. Audiogram showed the bilateral severe deafness (the degree of hearing as follow; normal: 0 dB to 30 dB, mild: 30 dB or more, moderate: 50 dB or more, severe: 70 dB or more, and deaf: 100 dB or more)

**Fig. 3:**
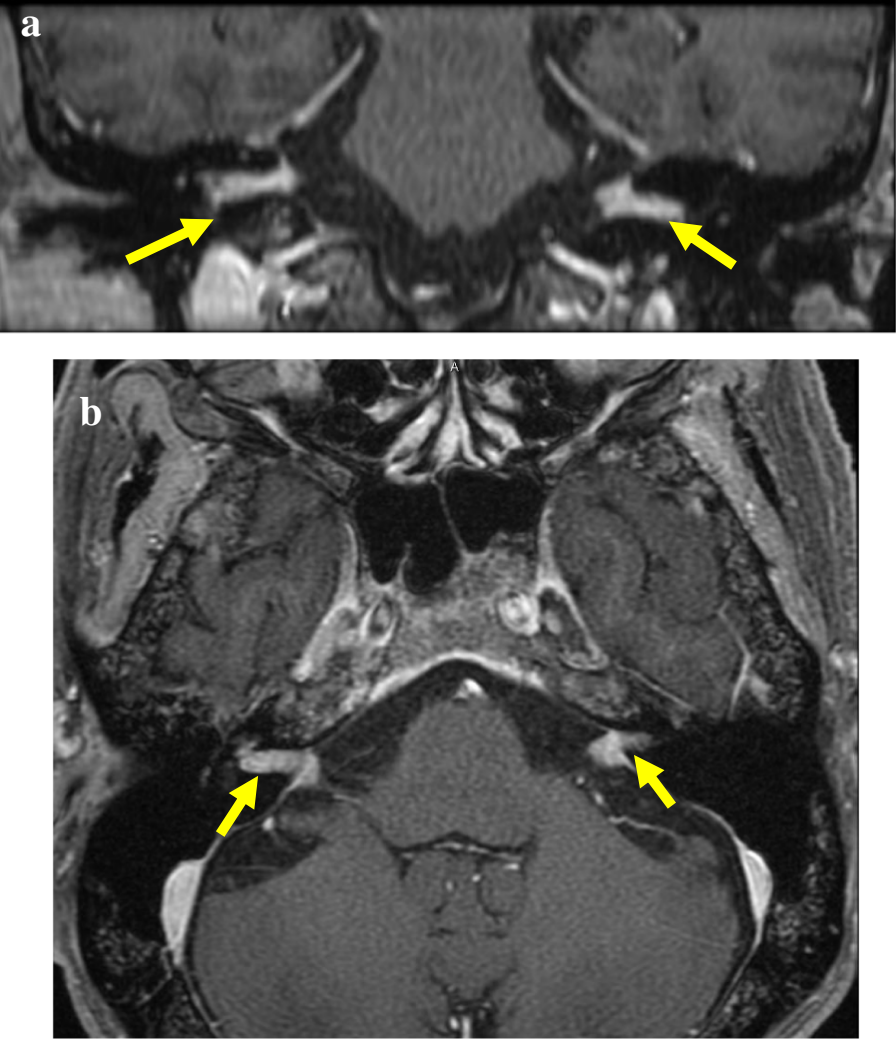
Enhanced MRI scan. **a** (coronal), **b** (horizontal): MRI scan showing the neoplastic lesion filling the bilateral internal auditory canal

**Fig. 4:**
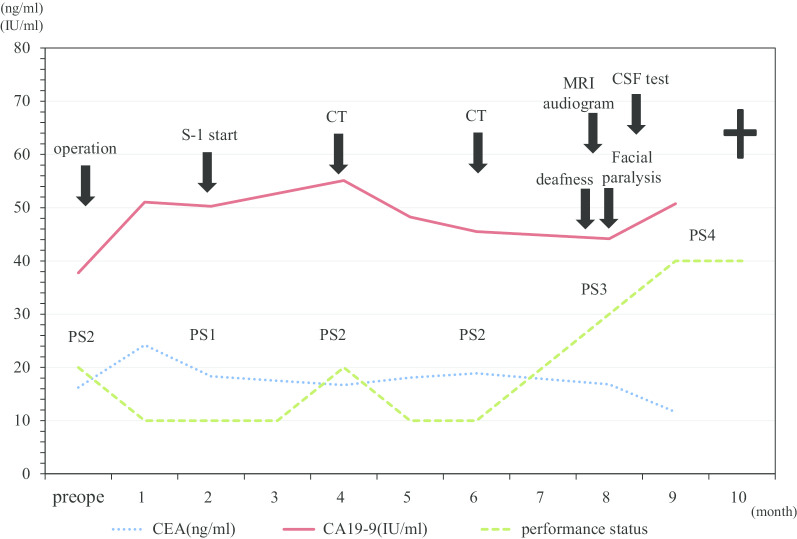
Clinical progression. Tumor marker levels remained high after performing the operation and administration of S-1 as adjuvant chemotherapy; the performance status rapidly got worse after neurological symptom appeared

A-68-year old man visited a hospital with vomiting and hematemesis. The laboratory test showed severe anemia. Therefore, he was referred to our hospital and laboratory tests were performed including the tumor markers. The tests revealed a WBC count of 8,980 cells/ml and Hb level of 9.1 g/dl, and BUN level of 23.6 mg/dl. CEA and CA19-9 levels were 16.23 ng/ml and 37.76 IU/ml, respectively. He underwent an emergency gastroscopy, which revealed Borrman type 3 tumor and oozing of blood (Fig. 1a). Therefore, biopsy was performed and he was diagnosed with gastric cancer (signet ring cell carcinoma). Afterwards, computed tomography (CT), colonoscopy, and upper gastrointestinal series revealed lymph node enlargement in the perigastric area and no distant metastasis (Fig. 1b). Under the clinical stage of T3N(+)M0, cStage III, he underwent total gastrectomy and Roux-en-Y reconstruction with D2 lymph node dissection. Although peritoneal lavage cytology was negative and liver metastasis and peritoneal carcinomatosis were not recognized in the operative findings, the tumor was exposed outside the serosa and No3 lymph node, which enlargement was preoperatively detected by CT, invaded the lesser omentum directly (Fig. 1c). Histopathologic findings were poorly differentiated adenocarcinoma with signet ring cell carcinoma (U, Ant, Type 3, 6 × 4.5 cm, por1 > sig, pT4a, INFc, Ly1b, V0, pPM0 (1.2 cm), pDM0 (12 cm), pN3b (18/44), M0, pStage IIIC). Tegafur-gimeracil-oteracil potassium (S-1) as adjuvant chemotherapy was initiated. However, the tumor marker levels remained high (CEA level, 24.20 ng/ml; CA19-9, 51.04 IU/ml) 3 months after the operation. Enhanced CT was performed and there was no recurrence in any area including the lung, liver, etc. and ascites was not recognized either. Therefore, we decided to follow up in the short period with continued adjuvant chemotherapy. Although the tumor marker level had remained high for a while, apparent recurrence was never detected despite short period CT. Nine months after surgery, he suddenly noted difficulty in hearing, and the deafness in both the ears progressed rapidly with facial paralysis; dizziness, tinnitus, and appetite loss worsened. Hearing test was also performed and audiogram revealed bilateral deafness (Fig. 2). Thus, cerebral infraction was suspected, and magnetic resonance imaging (MRI) was performed. MRI showed a neoplastic lesion filling the bilateral internal auditory canal, although the MRI did not show any evidence of cerebral infraction and meningeal hyperplasia, and dissemination was not recognized (Fig. 3a, b). Finally, cerebrospinal fluid (CSF) test was performed and adenocarcinoma was detected in the CSF using the Papanicolaou stain, and he was diagnosed with meningeal carcinomatosis. Figure 4 describes the progression of his situation including the tumor markers levels. After confirming meningeal carcinomatosis, radiotherapy or chemotherapy was planned. However, his situation deteriorated rapidly and he could receive neither radiotherapy nor chemotherapy because of the rapid deterioration of his performance status. He died approximately two months after the symptom onset.

## Discussion

Meningeal carcinomatosis occurs when cancer cells diffusely infiltrate the cerebrospinal cavity and form metastatic foci [10]. Particularly, meningeal carcinomatosis within the internal auditory canal (IAC) is relatively rare and its prognosis is very poor [8, 10]. Chang and Michaelidis reviewed some literature regarding meningeal carcinomatosis of the IAC and reported that 52.9% of 102 cases were bilateral. They also reported that the lung (21.2%), skin (18.6%), and breast (16.7%) were the sites of primary origin in bilateral cases and that the rate of co-occurring sites of metastatic disease present in cases of meningeal carcinomatosis within IAC was over 60% [10, 11]. Other studies reported that the incidence of meningeal carcinomatosis in gastric cancer is 0.1 ~ 0.69% [4–7]. Despite the fact that tumor markers remained high in this case, CT could not detect the recurrence in the whole body. Therefore, meningeal carcinomatosis within IAC was considered to be the first recurrence site for this patient.

In general, meningeal carcinomatosis shows several symptoms such as hearing loss, headache, nausea, vertigo, etc. When a cranial nerve symptom appears, the II, III, VII, and VIII cranial nerves are injured, and the VII and VIII cranial nerves are particularly injured [12]. It was reported in the mechanism of onset that cancer cells infiltrate the cerebrospinal fluid, the cerebrospinal fluid stagnates in the basal cistern and the cerebellopontine angle, and the cancer cells easily spread in that area [12]. Other diseases that exhibit the bilateral hearing loss and vestibulopathy are shown in primary and metastatic brain tumor, neurofibromatosis II, Guillain-Barré syndrome, Fisher syndrome, etc [9]. In this case, after the appearance of symptoms related to the VIII nerves, the VII neuropathy appeared rapidly. However, no characteristic family history or rash findings were observed, and no prior suspected infection of Guillain-Barré syndrome or Fisher syndrome was observed. Therefore, primary brain tumor, metastatic brain tumor, or brain infraction was suspected.

Enhanced MRI is reported to be very effective in diagnosing meningeal carcinomatosis. Some studies reported that the sensitivity of enhanced MRI for meningeal carcinoma is approximately 70% and is more effective than enhanced CT, the sensitivity of which is approximately 30% [13, 14]. Additionally, to obtain a definitive diagnosis, CSF test is the most effective. CSF protein is increased in 75%, CSF pressure is increased in 50%, and glucose decrease in CSF is observed in 40% of cases [15, 16]. However, the positive rate of cerebrospinal fluid in the initial test is approximately 45–50% and CSF tests should be performed multiple times if meningeal carcinomatosis is suspected [15, 16]. In this case, enhanced MRI revealed the neoplastic lesion filling the bilateral internal auditory canal and CSF test showed class V (adenocarcinoma). Meningeal carcinomatosis due to gastric cancer was confirmed.

No standard treatment has been clearly defined for the management of meningeal carcinomatosis. The treatment for meningeal carcinomatosis is aimed at restraining or improving the neurological symptom and extending the survival. The main treatment for meningeal carcinomatosis is chemotherapy. However, drug delivery into the intrathecal cavity is reported to be difficult with systemic chemotherapy due to blood–brain barrier [17, 18]. Therefore, the intrathecal chemotherapy with Methotrexate or Cytarabine or whole brain irradiation is often attempted in patients with stable general condition or stable performance status [19]. Rapid introduction of the intrathecal chemotherapy is reported to be effective in alleviating the neurological symptom [20]. However, no large-scale study has evaluated its efficacy, and further studies on the administration period are necessary. In this case, his condition and performance status rapidly got worse after confirmed diagnosis. Therefore, the intrathecal chemotherapy or whole brain irradiation could not be introduced and the symptom could be reduced to the end.

The prognosis of meningeal carcinomatosis is very poor and the average survival period after diagnosis is reported to be approximately one month in untreated patients, two months in refractory patients and the duration of survival time depends on the primary tumor [21]. Our patient died approximately two months after the symptom onset and about one month after the confirmed diagnosis.

## Conclusion

It was difficult to detect the recurrence of our patient by general systemic examination despite the sustained high tumor markers and work up could not detect any metastatic site such as the liver and lung. Therefore, both CT and enhanced MRI should be performed to check the intracranial metastatic target in cases where the tumor marker levels remain high. If meningeal carcinomatosis is suspected, CSF test should be performed rapidly. If the diagnosis can be made quickly, the patient's symptom and distress might be alleviated, even though the prognosis is poor.

## Data Availability

All data generated or analyzed during this study are included in this published article.

## Abbreviations

S-1: Tegafur-gimeracil-oteracil potassium
IAC: Internal auditory canal
CT: Computed tomography
MRI: Magnetic resonance imaging
CSF: Cerebrospinal fluid
